# ‘Nature or nurture’: survival rate, oviposition interval, and possible gonotrophic discordance among South East Asian anophelines

**DOI:** 10.1186/s12936-016-1389-0

**Published:** 2016-07-12

**Authors:** J. Derek Charlwood, Somalay Nenhep, Siv Sovannaroth, John C. Morgan, Janet Hemingway, Nakul Chitnis, Olivier J. T. Briët

**Affiliations:** Liverpool School of Tropical Medicine, Pembroke Place, Liverpool, L3 5QA UK; CNM, Phnom Penh, Cambodia; Swiss Tropical and Public Health Institute, Socinstrasse 57, 4002 Basel, Switzerland; University of Basel, 4003 Basel, Switzerland; PAMVERC, P.O. Box 10, Muleba, Tanzania

## Abstract

**Background:**

Mosquito survival, oviposition interval and gonotrophic concordance are important determinants of vectorial capacity. These may vary between species or within a single species depending on the environment. They may be estimated by examination of the ovaries of host-seeking mosquitoes.

**Methods:**

Landing collections, Furvela tent-trap and CDC light-trap collections were undertaken sequentially in four locations in Cambodia between February 2012 and December 2013 and samples from the collected mosquitoes were dissected to determine parity, sac stage (indicative of time spent prior to returning to feed) and egg stage.

**Results:**

A total of 27,876 *Anopheles* from 15 species or species groups were collected in the four locations and 2883 specimens were dissected. Both the density and predominant species collected varied according to location and trapping method. Five species were dissected in sufficient numbers to allow comparisons between locations. Estimated oviposition interval differed markedly between species but less within species among different locations. *Anopheles aconitus* had the shortest cycle, which was 3.17 days (95 % CI 3–3.64), and *Anopheles barbirostris* had the longest cycle, which took four days (95 % CI 3.29–4). *Anopheles minimus* had a higher sac rate in weeks leading up to a full moon but there was apparently little effect of moon phase on *Anopheles dirus*. Despite the fact that many of the species occurred at very low densities, there was no evidence of gonotrophic dissociation in any of them, even during sustained hot, dry periods. The principal Cambodian malaria vector, *An. dirus*, was only common in one location where it was collected in miniature light-traps inside houses. It did not appear to have an exceptional survival rate (as judged by the low average parous rate) or oviposition cycle.

**Conclusions:**

Differences in the oviposition interval were more pronounced among species within locations than within species among ecologically diverse locations. A nationwide survey using CDC light-traps for the collection of *An. dirus* inside houses may help in determining patterns of malaria transmission in Cambodia.

**Electronic supplementary material:**

The online version of this article (doi:10.1186/s12936-016-1389-0) contains supplementary material, which is available to authorized users.

## Background

The vectorial capacity of a physiologically competent mosquito population to transmit malaria is, amongst others, affected by population density, survival rate, infection rate, oviposition interval (blood-feeding frequency), time and location of biting, and host preference. In particular, the expected infective life (i.e., the average number of potentially infective bites of a female mosquito during its lifetime) is a function of vector survival rate and oviposition interval. Since activities such as blood feeding and oviposition carry more risks for the mosquito than post-prandial resting, mosquito survival is probably most accurately expressed per entire oviposition cycle than per day [1]. In contrast, pathogen development depends on ambient temperature and progresses daily. Assuming a similar survival rate per cycle, infected mosquitoes that have a long cycle are, therefore, more likely to survive to become vectors than mosquitoes with shorter cycles.

A number of factors may influence the duration of the oviposition cycle. Although low temperatures may delay oogenesis and thus act as a physiological constraint [2], in the tropics, the constraints are more likely to be ecological. For example, post-ovipostion behaviour in *Anopheles farauti* from Papua New Guinea is largely determined by environmental conditions; in particular the moon phase [3, 4] and distance to the oviposition site [5]. Obsomer and colleagues [6] also indicate that moon phase affects the duration of the oviposition cycle of *Anopheles dirus* in a similar manner. This may be due to changes in the time it takes before a mosquito returns to feed following oviposition. Cycle length for *Anopheles funestus* from Mozambique has been observed to vary with infection status, but whether this variation is due to ‘manipulation’ by the parasite or due to non-adaptive, coincidental side effects of parasite infections is unknown [7].

Of the many *Anopheles* biting humans in Cambodia, only a few are vectors of malaria and only *An. dirus* is a vector of any consequence [8]. Differences in the duration of the oviposition cycle, due to differences in post-ovipostion behaviour, and differences in survival may be responsible for disparity in vectorial capacity between species. Post-oviposition behaviour has, however, not previously been studied among S.E. Asian mosquitoes and even studies determining survival rates are limited [9, 10 quoted in 11].

Another determinant of vectorial capacity is the number of blood meals taken per gonotrophic cycle. Anophelines are generally gonotrophically concordant. In other words, they have a regular cycle in which each blood meal results in an egg batch laid the night that the mosquito becomes completely gravid. However, under conditions of environmental stress, especially during hot and dry conditions when potential oviposition sites dry up, some species may delay oviposition and take several feeds during a cycle, which can then take weeks or months, instead of days, to complete [12–15]. So called, gonotrophic discordance was described more than 50 years ago among *Anopheles maculatus*, *Anopheles culicifacies*, *Anopheles annularis* and *Anopheles aconitus* from India and Southeast Asia [16–18]. Since they are so long lived, even low-density populations of gonotrophically discordant mosquitoes are likely to be epidemiologically dangerous.

Gonotrophically discordant females have ovarioles at an advanced stage of development, and many are gravid when they (re)feed [13], whilst gonotrophically concordant females have relatively undeveloped ovarioles. Examination of the females’ ovaries, therefore, allows for detecting the presence of gonotrophically discordant females, as well as the parous rate (the proportion of females that have previously laid eggs) and, among parous females, for an estimation of the time taken to return feed following oviposition.

As a result of deforestation, hot, dry environments of the sort where gonotrophically discordant mosquitoes might be found are now common for much of the year in Cambodia. The phenomenon has, however, not been investigated in recent years. One way of determining if post-oviposition behaviour is environmentally determined and if populations become gonotrophically discordant in response to environmental stress, is to examine the behaviour of the same species of mosquito sampled from a variety of locations over a reasonable length of time. The ovaries of host seeking *Anopheles* caught while attempting to feed on people or domestic animals from four locations in Cambodia (three of which, at different times, were hot and dry) were examined to see if species groups showed evidence of being gonotrophically discordant and if mosquito parous rates and post oviposition interval differed among locations. In those species collected in sufficient numbers for analysis the possible effect of moon phase on the duration of the cycle was also determined.

## Methods

### Description of study locations

**Fig. 1:**
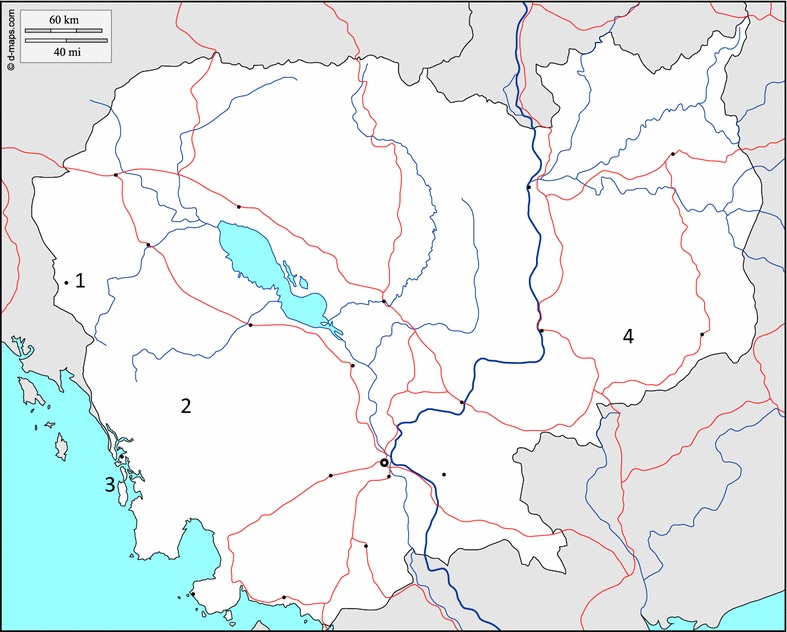
Map of Cambodia showing the four locations where collections and dissections were undertaken during the study. *1* Khum Otavao, Pailin Province; *2* Krorhom Krom, Veal Veng, Pursat Province, *3* Kroh Salau, Koh Khong Province; *4* Ou Chra, Mondolkiri Province

Collections of host seeking mosquitoes, caught by a variety of methods, were undertaken between February 2012 and December 2013 in the four locations shown in Fig. 1 and described below.

### Khum Otavao, Pailin Province

Much the greater part of the work was undertaken in Khum Otavao (N12.789 E102.690), a village on a shallow ridge 15 km from Pailin town. Pailin Province is the centre of delayed *Plasmodium falciparum* clearance by artemisinin [19]. Historically, Pailin Province was densely forested, and malaria transmission was relatively intense. In recent years, the province has been deforested and transmission has decreased markedly [20]. The natural forest cover in a radius of 2 km round the collection site reduced from 9.8 % in 2010 to 3.6 % in 2015 [21]. People grow cassava as a cash crop. In Khum Otavao, a man-made pond is used to store water for use during the dry season. During the rainy season, jars and pots are used to collect rainwater close to houses. A small seasonal stream, that tends to become a marsh in the wet season, runs to the west of the village.

### Krorhom Krom, Veal Veng, Pursat Province

In the study location Krorhom Krom (N12.215 E 103.080), 25 km out of Veal Veng town (Pursat Province), people live in isolated, separate houses built alongside the road (much as people do in the Amazon). They grow maize as a cash crop. In the dry season, much of the vegetation in the area is burnt. Pigs, buffalo, cows and dogs are common in Krorhom Krom. Active deforestation was ongoing in the area and reductions in forest cover were visible between study visits. The natural forest cover in a radius of 2 km round the collection site reduced from 99.9 % in 2010 to 70.1 % in 2015 [21]. Pursat Province is also an area where malaria parasites are being cleared slowly when treated with artemisinin [22].

### Kroh Salau, Koh Khong Province

The village of Kroh Salau (N 11.460 E 103.049) is on an island in the Krasaop Wildlife Sanctuary, Koh Kong Province. The island is surrounded by mangrove forest but rises to ≈200 m above sea level where there is a pagoda. The natural forest cover in a radius of 2 km round the collection site reduced from 46.8 % in 2010 to 0 % in 2015 [21]. Fishing is the main occupation. The village consists of numerous closely packed wooden houses on stilts either built over the water or close to the shore. Dogs are reasonably common but other domestic animals are rare.

### Ou Chra and Pu Cha, Mondolkiri Province

The village of Ou Chra (N 12. 237, E106.848) is situated in a rice growing area, which, at the time of the study, was still surrounded by secondary forest [23]. The village consists of 32 houses made of wood or bamboo, many of them on stilts. The larger village of Pu Cha lies 5 km to the north of Ou Chra. The two villages are separated by secondary forest. The 52 houses closest to Ou Chra were used during the study. Pigs, buffalo and dogs are common in both villages. Active logging and forest clearance was also ongoing in Ou Chra. Nevertheless, the forest remained closer to the village than in the other locations. The natural forest cover in a radius of 2 km around Ou Chra reduced from 98.0 % in 2010 to 77.4 % in 2015 [21]. People also used the forest for hunting and collection of more sustainable resources. They were the only two study villages where *An. dirus* was relatively common and where active malaria transmission was occurring.

With the exception of Ou Chra, all collection sites were areas where work, in particular landing collections, by the National Centre of Malariology (CNM) had previously been undertaken [8].

### Mosquito collection

Landing collections were the main collection method used in Khum Otavao (Pailin), Kroh Salau (Koh Kong) and Krorhom Krom (Pursat). Landing collections, performed by eight collectors, were largely undertaken for the first 4 h after sunset although all night collections were also performed in Khum Otavao (Pailin), where six all night collections were done during March–April 2012 compared to 179 4-h collections. Mosquitoes were collected with an aspirator as they landed on the exposed lower legs of adults. In Kroh Salau (Koh Kong), where autochthonous malaria transmission no longer occurred, young women as well as men collected. All collectors had done such work previously.

Landing collections were not performed in Ou Chra, due to the inherent risk involved in this area where autochthonous malaria transmission was possible. The principal mosquito collection method used in Ou Chra and Pu Cha was CDC light-traps hung close to sleepers (themselves under mosquito nets), indoors. Collections included on average 15 light-trap nights per house plus 49 light-trap nights from a sentinel house.

In order to obtain estimates of host preference among the local *Anopheles* in Krohm Otavao (Pursat), and Ou Chra (Monolkiri) light-traps were also hung close to pigs, cattle or buffalo and people sleeping outside on a platforms 1 m off the ground under houses. Mosquito Magnet (MMX) traps were also used in Pailin and Ou Chra (Mondolkiri). The traps burn liquid petroleum gas (LPG) to generate carbon dioxide and heat that attract mosquitoes, whilst at the same time powering a fan that sucks mosquitoes into a collection bag, where they die of dehydration [24]. As recommended by the manufacturers for every month’s use, an octanol attractant was installed in the trap. In total, the mosquito collections were performed during 185 nights in Pailin, 66 nights in Veal Veng, 43 nights in Kroh Salau and 114 nights in Ou Chra.

### Environmental data

Moon phase was divided into 4 weekly units that culminated in a full moon (i.e. the full moon phase consisted of the seven nights up to and including the full moon. Waning consisted of the subsequent seven nights, No moon the subsequent seven nights and waxing of the final seven nights that make up a complete lunar cycle). No assessment of cloud cover or of actual illumination was performed. Monthly weather data (at a spatial resolution of 0.5 × 0.5 degrees) for 2012 and 2013 was obtained from the CRU TS v3.23 dataset [25].

### Mosquito processing

Collected mosquitoes were identified morphologically using the keys of Rattanarithikul and Panthusiri [26] and Rattanarithikul and colleagues [27] to species or species complex. A small number of mosquitoes belonging to known complexes were identified by PCR using the PCR–RFLP. A sample of the mosquitoes morphologically identified as *Anopheles minimus* s.l. was identified using a PCR–RFLP, as described by Van Bortel and colleagues [28], based on the amplification of ITS2 rDNA region using primers ITS2A and ITS2B followed by a restriction using the Sau 961 restriction enzyme. Similarly, a sample of the mosquitoes morphologically identified as *An. maculatus* s.l. was identified using a PCR–RFLP, based on the amplification of ITS2 rDNA region using primers ITS2A and ITS2B as described by Durnez and colleagues [8], followed by a restriction using the HaeIII restriction enzyme complex. A random sample of the mosquitoes morphologically identified as *An. dirus* were identified using the PCR-protocol as described by Walton and colleagues [29].

Each hour’s collection was kept in individual containers and a sample of insects from the first 4 h of landing collection (18:00–21:00) was dissected in eye-drops (Optrex) with a dissection microscope with a mirror stage. By changing the angle of the mirror, the light that passed through the specimen was periodically altered so that the amount of contrast in the preparation could be maximized enabling visualization of the finer, more transparent structures. It also reduced dissector fatigue. The last two segments of those mosquitoes that remained flexible at the time of dissection were gently separated from the rest of the abdomen using minuten dissection needles, one of which was anchored the body to the slide whilst the other pulled the segments apart. The ovaries and intestines (including the Malphigian tubes and stomach) generally remained attached to these segments. If they broke off during the process of separation then further manipulation was required up to the point at which at least one of the ovaries had detached from the body and was available for more intimate dissection. The ovaries were separated from the other internal organs and teased apart while being examined at, circa ×40, through the dissection microscope.

The great majority of mosquitoes were, however, dead and dry upon examination. Dead insects are much less elastic than fresh insects and ovaries cannot be extracted in the usual manner. In order to dissect dry mosquitoes, the abdomen was punctured with needles so that the saline solution entered and partially re-hydrated the internal organs. After being left for a few minutes, an ovary or part of an ovary was teased out from the side of the abdomen and the state of the follicles determined. However, it was not possible, with any confidence, to determine the presence of mating plugs or spermatozoa in the spermatheca from such insects.

Females were separated into the following classes:

*Nulliparous I* Ovarioles at Stage I, with tracheoles visibly coiled and the ovary small and transparent. If freshly killed, females were also examined for the presence of mating plugs in the common oviduct. These mosquitoes were searching for their first blood meal when caught.

*Nulliparous II* Ovarioles at Stage II, (i.e., with some yolk present in the developing follicle) the ovary transparent and clean. These mosquitoes may have been searching for their first or second blood meal when caught.

*Parous with a*-*c sacs* The ovariolar stalk distended, indicating that the mosquito had returned to feed shortly after oviposition. Ovaries and oviducts with colour, and tracheoles not visible. From the middle of the study period onward, sacs were further classified into ‘a’, ‘b’ or ‘c’ sac subclasses according to the scheme given by Wilkes and Charlwood [30].

*Parous with d sacs* The sac from the previous oviposition contracted, indicating that there had been a delay between oviposition and returning for re-feeding. Ovaries and oviducts with colour, and tracheoles not visible.

The presence of retained Stage V eggs in parous females, according to the sac stage, was also noted. The one advantage of having dead mosquitoes to deal with was that the sac stage would have been that of the mosquito close to the time of collection rather than dissection (which may be 12 h later, ample time for the sac to contract from ‘a’ to ‘c’).

### Data analysis

Data analysis was performed in the R software environment [31]. Generalized linear mixed effect modeling was done using the R package lme4.

Differences in sac rate between species, or species group, within locations and between locations within species were calculated with Fisher’s exact test. With *A* the sac rate, or the proportion of parous host searching mosquitoes that laid eggs earlier that day, out of all parous host seeking mosquitoes and with *τ* the length of the gonotrophic cycle of a mosquito (from blood feeding to the start of searching for a new blood meal) and with some further assumptions, *u*, the average length of the feeding cycle in a mosquito population can be estimated.

Assuming that all mosquitoes succeed in blood feeding when host searching, but that some mosquitoes (*A*) take an extra day to rest, $u_{1} = \tau + (1 - A)$.

Assuming that all mosquitoes search for a host after *τ* days of resting, but have a daily probability of $P_{A} = 1 - A$ of surviving a day of host searching but not finding a host, $u_{2} = \theta_{f} = \tau + \frac{{P_{A} }}{{1 - P_{A} }} = \tau + \frac{1 - A}{A}$ (Chitnis et al. [32], Equation 23).

No information was collected on the value of *τ* but assumed a value of 3 days. Confidence intervals for *A* and *u*_1_ (due to uncertainty in *A*, but not taking uncertainty in *τ* into account) were calculated with Fisher’s exact test. Confidence intervals for *u*_2_ (due to uncertainty in *A*, but not taking uncertainty in *τ* into account) were estimated with a simple Bayesian model (Additional file 1).

## Results

### Species composition

All 102 *An. dirus* s.l. identified to species were *An. dirus* s.s. Therefore, it was presumed that this was the only member of the complex present. Unfortunately, only a very small number of mosquitoes from other species groups were identified to species by PCR. Although these numbers are very limited, the ratios obtained for both *An. minimus* s.l. and *An. maculatus* s.l. were similar to those of Durnez and colleagues [8], who identified 544 specimens from the same study sites in 2007 (Fishers exact statistics comparing the most common species identified and all other species for both *An. minimus* s.l. and *An. maculatus* s.l. were not significant at the 0.05 % level). Thus, *An. minimus* was the most common member of the complex collected whilst *Anopheles sawadwongporni* predominated among the *An. maculatus* complex. Nevertheless, the data were amalgamated according to species group or complex for subsequent analysis. Another mosquito that is difficult to separate morphologically from *An. minimus* s.l. is *An. aconitus*. The *An. aconitus* dissected had fewer follicles than the *An. minimus* s.l. and were largely collected in tent traps away from the village. Hence it was considered as a separate species. The number of *Anopheles* collected are given in Table 1. A total of 15 species or species groups of *Anopheles* were morphologically identified from the four study locations. Thirteen species or species complexes of anophelines were identified from light-trap collections in Ou Chra and Pu Cha, and six from light-trap collections in Pailin. *Anopheles minimus* s.l. was the most common mosquito in Khum Otavao (Pailin) and *An. maculatus* s.l. predominated in Krorhom Krom (Pursat). *Anopheles hodgkini* was the predominant one of the seven species collected on the island of Kroh Salau (Koh Kong), while the only place where *An. dirus* predominated was Ou Chra (Mondolkiri) (Table 1).

**Table 1:** Number of mosquitoes caught in sites by species and method

Location	Collection	*An. minimus* s.l.	*An. maculatus.* s.l.	*An. barbirostris* s.l.	*An. aconitus*	*An. kochi*	*An. umbrosus*	*An. philippinensis*	*An. dirus* s.l.	*An. vagus*	*An. jamesii*	*An. tessellatus*	*An. karwai*	*An. hodgkini*	*An. sinensis*	*An. epiroticus*	Total anophelines
Pailin	Landing	6747	1763	518	509	0	4	41	12	44	18	325	0	0	0	0	9981
	Light-trap (outdoor)	583	245	5	75	0	0	0	0	17	0	48	0	0	0	0	973
	Tent-trap	1151	112	76	186	0	0	8	1	13	4	168	0	0	0	0	1719
	MMX trap	160	20	82	183	0	1	6	0	82	3	9	0	0	0	0	546
Veal Veng	Landing	172	2238	119	309	1986	2	40	27	199	909	39	2	0	0	0	6042
	Light-people	16	145	0	11	111	0	0	3	2	6	0	0	0	0	0	294
	Light-cow	17	573	5	8	568	0	0	0	0	18	1	0	0	0	0	1190
	Light-pig	21	933	26	15	1708	0	0	0	1	30	2	0	0	0	0	2736
Koh Kong	Landing	0	0	59	0	3	91	0	0	0	1	0	7	1231	459	185	2036

The mean number of mosquitoes collected according to host from Krorhom Krom (Pursat) is shown in Table 2. Each species, or species complex, was caught in greatest numbers in the trap hung close to the three (little) pigs. This was particularly true for the *Anopheles kochii*.

**Table 2:** Mean number of *Anopheles* species collected in light-traps hung adjacent to different hosts outdoors, Veal Veng, Pursat, Cambodia

*Species*	Host		
	Two humans	Two cows	Three (little) pigs
*An. aconitus*	0.42	0.24	0.45
*An. minimus*	0.62	0.59	0.72
*An. maculatus*	5.58	19.72	32.10
*An. kochi*	4.27	19.59	58.90
*An. jamesi*	0.23	0.62	1.00
*An. barbirostris*	0.00	0.17	0.72

*Anopheles kochii* also predominated (67 %) in light-traps placed close to a pig sty in Ou Chra. Although *An. dirus* was the most common mosquito inside houses in Ou Chra, accounting for 74 % of all anophelines collected, it was only the ninth most common species collected from light-traps hung close to pigs. It was the least common of the nine species collected in the MMX trap (in this case *Anopheles barbirostris* s.l. was the most common species accounting for 69 % of the collection).

### Dissection results

Altogether, 2883 mosquitoes from 14 species and species groups were dissected (Additional file 2). Table 3 shows the parous and sac rates, with derived estimates of the oviposition intervals of the species dissected, according to the location and method of collection. Overall, *An. aconitus* had the shortest [3.17 days (95 % CI 3–3.64)] and *An. sinensis* the longest [4.0 days (95 % CI 3.29–4)] estimated cycles. The age of dissected females was independent of trapping method.

**Table 3:** Parous rates, sac rates and cycle length for species and sites

Site	Species		Dissected non-gravid	Parous	Parous rate (95 % CI)	Sac rate (95 % CI)^a^	Cycle length (u1)	Cycle length (u2)
Mondolkiri	barbirostris s.l.	An	24	20	0.83 (0.63–0.95)	0.2 (0.06–0.44) a	3.8 (3.56–3.94)	7 (4.39–14.28)
Koh Kong	barbirostris s.l.	An	4	3	0.75 (0.19–0.99)	0 (0–0.71) a	4 (3.29–4)	NA
Pailin	barbirostris s.l.	An	72	36	0.5 (0.38–0.62)	0.14 (0.05–0.29) a	3.86 (3.71–3.95)	9.2 (5.51–18.19)
Pursat	barbirostris s.l.	An	19	7	0.37 (0.16–0.62)	0.29 (0.04–0.71) a–b	3.71 (3.29–3.96)	5.5 (3.54–13.99)
Mondolkiri	hodgkini	An	5	0	0 (0–0.52)	NA	NA	NA
Koh Kong	sinensis	An	77	19	0.25 (0.16–0.36)	0.16 (0.03–0.4) a	3.84 (3.6–3.97)	8.33 (4.64–19.72)
Koh Kong	umbrosus	An	2	0	0 (0–0.84)	NA	NA	NA
Pailin	umbrosus	An	2	0	0 (0–0.84)	NA	NA	NA
Mondolkiri	aconitus	Ce	9	6	0.67 (0.3–0.93)	0.83 (0.36–1) a–b	3.17 (3–3.64)	3.2 (3.04–4.39)
Pailin	aconitus	Ce	42	25	0.6 (0.43–0.74)	0.48 (0.28–0.69) a–b	3.52 (3.31–3.72)	4.08 (3.5–5.34)
Pursat	aconitus	Ce	33	25	0.76 (0.58–0.89)	0.32 (0.15–0.54) a	3.68 (3.46–3.85)	5.13 (3.93–7.84)
Mondolkiri	dirus s.l.	Ce	379	220	0.58 (0.53–0.63)	0.34 (0.27–0.4) a	3.66 (3.6–3.73)	4.97 (4.5–5.62)
Pailin	dirus s.l.	Ce	5	2	0.4 (0.05–0.85)	0.5 (0.01–0.99) a–b	3.5 (3.01–3.99)	4 (3.1–12.53)
Pursat	dirus s.l.	Ce	11	2	0.18 (0.02–0.52)	1 (0.16–1) a–b	3 (3–3.84)	3 (3.01–5.46)
Koh Kong	epiroticus	Ce	137	40	0.29 (0.22–0.38)	0.33 (0.19–0.49) a	3.68 (3.51–3.81)	5.08 (4.09–6.96)
Mondolkiri	jamesii	Ce	1	1	1 (0.03–1)	1 (0.03–1) a–b	3 (3–3.98)	3 (3.01–8.29)
Pailin	jamesii	Ce	1	0	0 (0–0.98)	NA	NA	NA
Pursat	jamesii	Ce	105	78	0.74 (0.65–0.82)	0.42 (0.31–0.54) a	3.58 (3.46–3.69)	4.36 (3.88–5.12)
Mondolkiri	kochi	Ce	53	35	0.66 (0.52–0.78)	0.6 (0.42–0.76) b	3.4 (3.24–3.58)	3.67 (3.34–4.31)
Pursat	kochi	Ce	300	215	0.72 (0.66–0.77)	0.74 (0.68–0.8) b	3.26 (3.2–3.32)	3.35 (3.26–3.48)
Mondolkiri	maculatus s.l.	Ce	5	3	0.6 (0.15–0.95)	0.33 (0.01–0.91) a–b	3.67 (3.09–3.99)	5 (3.24–16.72)
Pailin	maculatus s.l.	Ce	232	122	0.53 (0.46–0.59)	0.52 (0.42–0.61) b	3.48 (3.39–3.58)	3.94 (3.66–4.33)
Pursat	maculatus s.l.	Ce	389	214	0.55 (0.5–0.6)	0.49 (0.42–0.56) a	3.51 (3.44–3.58)	4.04 (3.8–4.35)
Pailin	minimus s.l.	Ce	835	568	0.68 (0.65–0.71)	0.6 (0.56–0.64) b	3.4 (3.36–3.44)	3.67 (3.56–3.79)
Pursat	minimus s.l.	Ce	27	21	0.78 (0.58–0.91)	0.48 (0.26–0.7) a–b	3.52 (3.3–3.74)	4.1 (3.48–5.53)
Mondolkiri	minimus s.l.	Ce	1	1	1 (0.03–1)	0 (0–0.98) a–b	4 (3.03–4)	NA
Mondolkiri	philippinensis	Ce	31	19	0.61 (0.42–0.78)	0.68 (0.43–0.87) b	3.32 (3.13–3.57)	3.46 (3.18–4.18)
Pailin	philippinensis	Ce	4	2	0.5 (0.07–0.93)	0.5 (0.01–0.99) a–b	3.5 (3.01–3.99)	4 (3.1–12.05)
Mondolkiri	tessellatus	Ce	5	1	0.2 (0.01–0.72)	0 (0–0.98) a–b	4 (3.03–4)	NA
Pailin	tessellatus	Ce	10	9	0.9 (0.55–1)	0.78 (0.4–0.97) a–b	3.22 (3.03–3.6)	3.29 (3.07–4.25)
Pursat	tessellatus	Ce	8	2	0.25 (0.03–0.65)	0 (0–0.84) a–b	4 (3.16–4)	NA
Mondolkiri	vagus	Ce	0	0	NA	NA	NA	NA
Pailin	vagus	Ce	12	5	0.42 (0.15–0.72)	1 (0.48–1) a–b	3 (3–3.52)	3 (3–3.83)
Pursat	vagus	Ce	25	23	0.92 (0.74–0.99)	0.39 (0.2–0.61) a	3.61 (3.39–3.8)	4.56 (3.69–6.52)

^a^Sac rates for species sharing a common letter were not significantly different within a province

In Ou Chra (Mondolkiri), sac rates were significantly lower for *An. barbirostris* s.l. and *An. dirus* s.l. as compared to *An. kochi* and *Anophelesphilippinensis*. In Pailin, sac rates were significantly lower for *An. barbirostris* s.l. as compared to *An. maculatus* s.l. and *An. minimus* s.l. In Krorhom Krom (Pursat), sac rates were significantly lower for *An. aconitus*, *Anopheles jamesi*, *An. maculatus* s.l. and *Anopheles vagus* as compared to *An. kochi*. Fisher’s exact tests did not detect significant differences in sac rates between locations for any of the species dissected in sufficient numbers for adequate comparisons. However, analysis of all data combined using generalized linear regression models of the binomial family detected that a model including a (positive) linear effect for the relative humidity in the month of capture and also fixed effects for location significantly (p = 0.0096, likelihood ratio test) improved model fit (deviance = 895.1), over a model with just random effect intercepts for species (deviance = 908.5). Therefore, there was an indication that apart from species-specific factors, the length of the oviposition cycle might vary with environmental factors.

Sac stages for 135 mosquitoes from eleven species dissected in 2013 are shown in Table 4. With the exception of *An. jamesii* and *An. barbirostris* s.l. (two of the species with the lowest rate of mosquitoes returning to feed with sacs), sacs were largely considered to be ‘a’ or ‘b’ (i.e. large to medium sized) indicating that the mosquitoes had returned to feed on the night following oviposition.

**Table 4:** The proportion of parous mosquitoes dissected by sac stage

Species	Proportion of parous with sacs	Sac stage		
		a	b	c
*An. aconitus*	0.43	2	0	2
*An. epiroticus*	0.33	0	1	0
*An. dirus* s.l.	0.34	22	12	11
*An. vagus*	0.50	1	0	0
*An. minimus* s.l.	0.60	15	18	8
*An. maculatus* s.l.	0.50	8	5	2
*An. kochi*	0.72	4	2	0
*An. philippinensis*	0.68	3	6	2
*An. tessellatus*	0.58	2	0	1
*An. jamesii*	0.42	0	2	5
*An. barbirostris* s.l.	0.20	0	0	1

In the week prior to a full moon, sacs rates in *An. minimus* s.l. from Pailin increased (indicating a shorter oviposition cycle) but moon phase did not appear to affect the duration of the cycle in *An. dirus* s.l. from Ou Chra (Mondolkiri) (Table 5).

**Table 5:** Number and proportion of *An. minimus* s.l. and *An. dirus* with sacs according to moon phase

Moon phase	*An. minimus* s.l.			*An. dirus* s.l.		
	With sacs	Without sacs	Proportion with sacs	With sacs	Without sacs	Proportion with sacs
New	152	97	0.61	–	–	–
First	91	88	0.51	37	66	0.36
Full	76	33	0.70	19	37	0.34
Last	32	21	0.60	21	44	0.32

Throughout the study period, at both wet and dry periods of the year, the great majority of insects were collected with ovaries at Christopher’s Stage I or Stage II. During all seasons (including the wet season when potential breeding sites were common) *An. vagus* females had ovaries at all stages of development including Stage III and Stage IV (Table 6). With the exception of *An. vagus*, the proportion of gravid insects among the other species dissected was always very low (Table 6). Females from rare species (i.e. where fewer than 10 individuals were collected) were not more likely to be gravid than females of the more common species.

**Table 6:** Proportion of mosquitoes dissected that were gravid on collection

Species	Total dissected	Number gravid	Best estimate (adjusted wald CI)
*An. aconitus*	85	1	0.023 (<0.0001–0.07)
*An. epiroticus*	138	1	0.014 (<0.0001–0.04)
*An. jamesii*	108	1	0.018 (<0.0001–0.06)
*An. maculatus* s.l.	632	6	0.011 (0.004–0.021)
*An. minimus* s.l.	865	2	0.004 (0.0001–0.009)
*An. vagus*	44	7	0.174 (0.076–0.297)

Females of the following species or species group were collected at least once with a mating plug [33]: *An. dirus* s.l. *An. maculatus* s.l., *An. minimus* s.l., *An. jamesi*, *An. aconitus*, *An. philippinensis* and *Anopheles tessellatus*. Females with mating plugs, in particular *An. maculatus* s.l., could often be recognized from the distension of the last segment of the abdomen, due to the plug, prior to dissection. At the height of the dry season in Ou Chra (Mondolkiri), one of three *An. dirus* s.l. dissected had Stage I ovaries and a mating plug indicating that breeding was still occurring at that time.

## Discussion

One of the biggest difficulties associated with working on the *Anopheles* fauna in S.E. Asia is the large diversity encountered and the large number of species complexes that occur there. Thus, among the fifteen species identified morphologically in the present study, several belong to complexes or morphologically identical groups. In samples previously identified from the same collection sites in Pailin and Veal Veng, one species of the *minimus* complex and one species of the *maculatus* group predominated (97 and 88 % of those identified by Durnez and colleagues [8]). Although only a small number of mosquitoes were identified by PCR in the present study, the ratios obtained were similar to those of the earlier study. Thus, although perhaps consisting of more than one member of each species complex, in particular in *An. minimus* s.l. and *An. maculatus* s.l., probably one member predominated. Closely related species are likely to have similar rather than extremely divergent behaviours. Since no differences were found within species between study sites in pair-wise comparisons, these results are likely to cover all members of the relevant species complexes or groups, even though the predominant member of any particular species group or complex may have differed between locations.

Densities of many species were very low. It is possible that the species collected in low numbers may be species that are not attracted to humans. Nevertheless, all of the species collected in light-traps close to cows or pigs were present in man-baited traps, albeit at different ratios. Given that the environment is rapidly changing in the study locations, it is also possible that we were witnessing the gradual demise of a number of species, including *An. dirus*, rather than rare species maintaining themselves by a set of effective survival strategies.

Sac rates (hence oviposition cycle duration) differed markedly between species, or species groups, while they were more similar within morphologically identified species or species groups across locations. Thus, intrinsic factors appeared to play a more important role in determining the time at which parous mosquitoes return to feed following oviposition, than extrinsic ones. Nonetheless, in regression analysis of data from all species and sites combined, relative humidity was positively associated with sac rate, and there were indications of differences between sites, with sac rates being higher in Pailin and Pursat compared to Mondolkiri. Also moon-phase appeared to affect the cycle in *An. minimus* s.l.: as with *An. farauti* from Papua New Guinea [4], it was shorter when moonlight was present prior to, or at, sunset, compared to nights when no moonlight was present at this time. Moonlight did not appear to affect the duration of the cycle in the forest dwelling *An. dirus* even though, in their review of factors affecting the biology of this species, Obsomer and colleagues [6] considered that moonlight was an important determinant of cycle duration. It is possible that, in the forest environment, where most ovipostion of *An. dirus* occurs, illumination is of less consequence than other factors in oviposition site and host location.

As Schapira and Boutsika [34] point out, S. E. Asian *Anopheles* are physiologically adapted to humid conditions, having wider spiracles than African ones. They may invest in large numbers of eggs per batch to the detriment of longevity, and thereby vectorial capacity. This is likely to reduce their ability to survive in hot dry environments, typically associated with gonotrophically discordant *Anopheles*. Indeed, the small number of gravid insects collected during the study implies that, for all species, including the least common ones, gonotrophic discordance was not the survival strategy adopted during the long, hot, dry season. Moreover, species such as *An. maculatus* s.l. and *An. aconitus*, previously reported as becoming discordant, did not show evidence of discordance. Thus, for all locations and all species, and at all times, gonotrophic concordance appeared to be the rule.

As far as we know, this is the first report of the presence of mating plugs in mosquitoes from S.E. Asia. The presence of an *Anopheles dirus* in Ou Chra with Stage I ovarioles and a mating plug, at the height of the dry season, when no standing water was seen in a number of protracted walks in the environs of the village, is an indication that active breeding was continuing, that both female and male insects were present and that gonotrophic discordance was unlikely to be occurring. Members of the complex are known to withdraw into the deep forest in the dry season, where they keep breeding, [35] and this may have been the case in Ou Chra.

*Anopheles minimus* s.l. was the most common mosquito in Khum Otavao, but despite the fact that it fed on humans and was relatively long-lived, it was not apparently a vector. No cases of malaria were reported from the village during the whole study period nor were any oocysts seen on the stomachs of dissected mosquitoes. This accords with the recent decline in malaria observed in Pailin province as a whole [20].

Ou Chra and Pu Cha were the only two villages where *An. dirus* predominated. *Anopheles dirus* was previously common in Pailin and Pursat (CNM unpublished data), but was rare or absent in the present study (only 27 specimens being caught in 2121 h of landing collection). Deforestation eliminates *An. dirus* and reduces malaria transmission. Indeed, the presence of members of the *An. dirus* complex is an indication of a healthy forest environment. It should, however, be possible to control malaria without destroying the forest.

Our results differ from those obtained elsewhere. The *An. dirus* were not exceptionally long lived (as indicated by Rosenberg and Maheswary [36], for populations in Pakistan), nor were they primarily zoophilic, as suggested by Gingrich and colleagues [35] and Tanachi [37] for populations from Thailand. Indeed, the results of collections close to animal baits or humans demonstrate the highly anthropophilic nature of this mosquito in this part of Cambodia. Thus, not only was it the most common mosquito collected close to humans inside houses but, in Ou Chra, it was the least common in the MMX trap and the ninth most common mosquito in traps over animals. Both its anthropophagic and endophilic behaviours (as well as being physiologically competent) are likely to be the cause of it being a malaria vector. Light-traps hung inside houses may, therefore, provide a suitable measure of *An. dirus* densities.

National malaria prevalence surveys are undertaken in Cambodia. These provide information on the groups of people most at risk from the disease but, because of the high rates of internal seasonal migration [38], do not provide information on the place where the infection was acquired. Given its unique role in malaria transmission a survey of vectors in Cambodia, using light traps inside houses, in particular in areas already deemed suitable for *An. dirus* by modelling studies [6], would seem reasonable and might help target control efforts.

## Conclusions

Although sac rates varied with relative humidity and moon phase (*An. minimus*), differences in oviposition interval were more pronounced among species within locations than within species among ecologically diverse locations. Despite rapid deforestation creating environments where one might expect gonotrophic discordance, low numbers of gravid host seeking females and the detection of mating plugs during the height of the dry season indicated that active breeding was continuing, albeit resulting in small populations. In contrast to findings from studies in Pakistan and Thailand, in this study, *An. dirus* showed anthropophagic and endophilic behaviour. Nationwide surveys using CDC light-traps inside houses may help in determining patterns of malaria transmission by *An. dirus* in Cambodia.

## Additional files

10.1186/s12936-016-1389-0 Bayesian model.
10.1186/s12936-016-1389-0 Data for dissected mosquitoes.

## Abbreviations

CDC: Centers for Disease Control and Prevention
CNM: National Centre of Malariology
CRU: Climatic Research Unit
ITS: internal transcribed spacer
LPG: liquid petroleum gas
LSTM: Liverpool School of Tropical Medicine
MMX: Mosquito Magnet X
PCR: polymerase chain reaction
rDNA: ribosomal deoxyribonucleic acid
RFLP: restricted fragment length polymorphism
S.E. Asia: Southeast Asia
TS: time series
